# Operational research highlights ongoing challenges for comprehensive TB services in Papua New Guinea

**DOI:** 10.5588/pha.24.0042

**Published:** 2024-09-01

**Authors:** A. Maha, T. Kelebi, A. Holmes, M. Kal, J. Greig, H. Nindil, S.M. Graham

**Affiliations:** ^1^ National Department of Health, Port Moresby, National Capital District, Papua New Guinea (PNG);; ^2^ West Sepik Provincial Health Authority, Vanimo, Sandaun Province, PNG;; ^3^ The Burnet Institute, Melbourne, VIC, Australia;; ^4^ University of Melbourne Department of Paediatrics, Royal Children’s Hospital, Melbourne, VIC, Australia.

## Abstract

Papua New Guinea (PNG) is a high-burden country for TB, with an estimated annual TB incidence rate of 432 per 100,000 population. There are major challenges to the provision of quality care for TB patients with high rates of loss to follow-up, and multidrug-resistant TB is increasingly detected. In 2022–2023, the second Structured Operational Research Training IniTiative (SORT-IT) for TB was undertaken. Eight participants completed the course, and the outputs from these research projects highlight important current operational issues for the PNG TB programme in a range of settings. The first four articles in the series are published in this issue of *Public Health Action,* with the remainder to follow in subsequent issues.

Papua New Guinea (PNG) is a high-burden country for drug-susceptible TB and for multidrug/rifampicin-resistant TB (MDR/RR-TB).^1^ In PNG, TB is a major cause of morbidity and mortality across all ages, and many individuals with TB remain undetected, and treatment success rates remain low. It is, therefore, critically important to improve both TB diagnosis and access to high-quality healthcare services. Operational research is fundamental to identifying implementation challenges for improved TB detection, treatment and prevention. The manuscripts included in this issue of *Public Health Action* present recent findings from SORT-IT projects undertaken by TB service providers in a range of settings in PNG. These add further perspective to the articles published in 2019 (Figure).^2^ Participants in SORT-IT courses acquire skills in research proposal development, data management, analysis and dissemination. Critically, the data acquired can be used to inform local solutions to help improve health service delivery. Furthermore, the acquired skills are relevant to a wide range of health programs beyond the TB programme.^3^ A wide range of topics are covered in this issue, including loss to follow-up in adults with drug-resistant TB;^4^ TB burden and diagnostic challenges;^5^ quality improvements in a community-wide TB screening and prevention program;^6^ and an analysis of unfavourable TB treatment outcomes.^7^ These present novel findings from a range of provinces in PNG (Figure).

**FIGURE.**
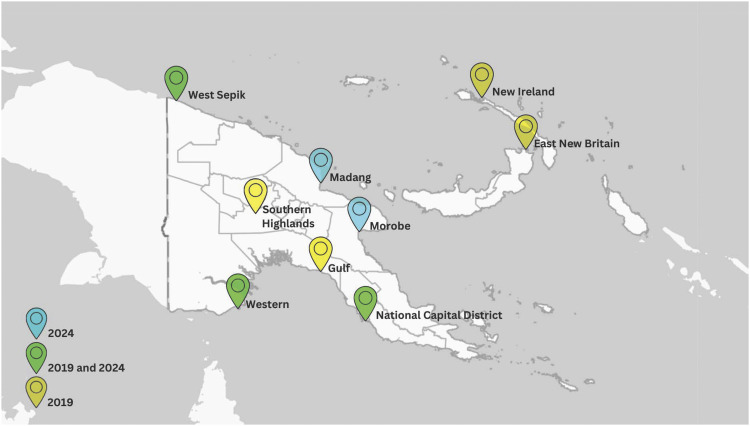
SORT-IT projects undertaken by TB service providers in a range of settings in PNG in 2019 and 2024. SORT-IT = Structured Operational Research Training IniTiative; PNG = Papua New Guinea.

Since GeneXpert implementation, MDR/RR-TB has increasingly been detected outside of the recognised ‘hot spot’ districts such as the National Capital District and the South Fly District of Western Province.^8,9^ The high rate of pre-treatment loss to follow-up for people diagnosed with MDR/RR-TB at PNG’s largest treatment centre is a major concern.^4^ This is especially problematic as it is common among young people who, without treatment, will suffer worse health consequences and also continue to transmit TB. The sustained high rates of treatment success for TB and MDR/RR-TB in the South Fly District demonstrate what can be achieved even in a setting with high rates of resistance to second-line drugs. However, this context of a small island population with established community engagement and treatment support mechanisms, as well as external funding support, is not representative of most settings in PNG.

Changes to diagnostic practices and spectrum over the past decade are also detailed.^5^ People with TB who live in rural or remote settings are underrepresented in these studies despite most of PNG’s population being rural-based. Clinical diagnosis is commonly required as access to laboratory diagnosis remains limited, and a high proportion of cases are childhood or extrapulmonary TB. Efforts to strengthen PNG’s diagnostic capacity continue. The shipment of samples abroad was found to be associated with contamination. Critically, wider access to resistance testing to a range of second-line drugs, including fluoroquinolones and ‘new’ drugs such as bedaquiline, is required for patient care and surveillance. Linezolid is an important and effective second-line drug, and although there is potential toxicity, original evidence is presented that it is well tolerated overall in people with MDR/RR-TB, in whom anaemia is common.

Although recommended, the implementation of contact investigation is extremely limited in PNG, despite its potential for detection and treatment of active TB while providing TB preventive treatment (TPT) for contacts without active disease. TPT guidelines are being updated with novel recommended regimens recommended. Evidence is being generated from community-based case-finding and treatment of disease and infection.^6^ Although this context has unique aspects, lessons learned will be informative and potentially widely relevant.

Finally, it is important to reemphasise that increased case detection and successful treatment of people with TB will reduce transmission and prevent new cases. A secure supply of drugs and access are major ongoing challenges in PNG. Uptake, adherence and completion remain critical for treating drug-susceptible or drug-resistant disease or infection.
